# Supplementary material for: Luminous Self-Assembled Fibers of Azopyridines and Quantum Dots Enabled by Synergy of Halogen Bond and Alkyl Chain Interactions
Source: Molecules. 2022 Nov 23;27(23):8165. doi: 10.3390/molecules27238165 (PMC9739974; doi:10.3390/molecules27238165)
Supplement: Supplementary file 1 [file molecules-27-08165-s001.zip › molecules-2001712-supplementary.pdf]

Supporting Information

Luminous Self-assembled Fibres of Azopyridines and Quantum Dots enabled by Synergy of Halogen Bond and Alkyl Chain Interactions

1. Synthesis of materials

The azopyridine compounds (AnAzPy) were synthesized through two steps of chemical reactions according to our previous study,[1] as shown in Scheme S1(a1)–(a2). Then halogen–bonded 15X (X: Br or I) complexes and 15X@QDs were synthesized as shown in Scheme S1(a3)–(a4).

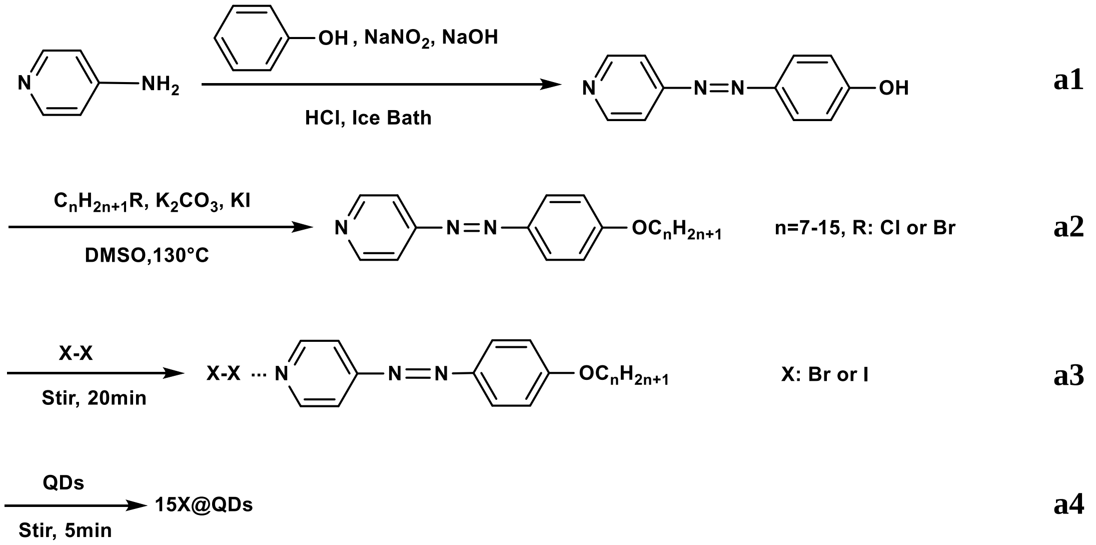

Scheme S1. Synthesis of the 15X@QDs.

1.1 Synthesis of 4–(–hydroxyphenylazo) pyridine (compound a1)

A 10 wt% NaOH aqueous solution (20 mL) containing sodium nitrate (4.00 g, 58 mmol) and phenol (5.00 g, 53 mmol) was prepared and cooled to 0 °C. Subsequently, it was added dropwise to another aqueous solution with HCl 45 mL (25 mL 11N HCl and 20 mL water) and 4–aminopyridine (6.00 g, 64 mmol). The reaction mixture was stirred under an ice bath (0 °C) for 0.5 h. Then, the pH of the reaction mixture was adjusted to pH=6–7 by addition of a 10 wt% NaOH aqueous solution. A yellow precipitate was collected by filtration. The crude product was washed with water and recrystallized from acetone. After drying over a vacuum for 24 h, the resulting bright yellow solid was obtained: yield 2.96 g (32.6%).

1.2 Synthesis of 4–(4–pentadecylphenylazo)pyridine (compound a2)

Taking A15AzPy as the typical representative: 1–bromopentadecane (2.91 g, 10 mmol) was dissolved in dimethyl sulfoxide (20 mL), which was added dropwise to a DMSO (20 mL) solution of K_2CO_3 (6.90 g, 5 mmol), KI (0.01 g, 0.1 mmol) and 4–(4–hydroxyphenylazo)pyridine (2.00 g, 10 mmol) at 130 °C. After 5 h, the mixture was poured into water (200 mL) and then extracted with ethyl acetate (50 mL×3). A rotary evaporator was used to remove all of the solvent. The crude product was purified by silica gel column chromatography with ethyl acetate as eluent, 2.84 g of the pure product was obtained as an orange powder. The ^1H NMR spectrum of compound a2 was recorded in 5 wt% $CDCl_3$ solution, as shown in Fig. 1. Yield: 58%. Mp: 80.7°C. ^1H NMR ($CDCl_3$) δ

8.77 (2H, d, Ar–H), δ 7.95 (2H, d, Ar–H), δ 7.71 (2H, d, Ar–H), δ 7.03 (2H, d, Ar–H), δ 4.06 (2H, t, –O–CH_2–), δ 1.85 (2H, m, –CH_2–), δ 1.46 (2H, m, –CH_2–), δ 1.27 (22H, m, –$C_{11}H_{22}$–), δ 0.90 (3H, t, –CH_3).

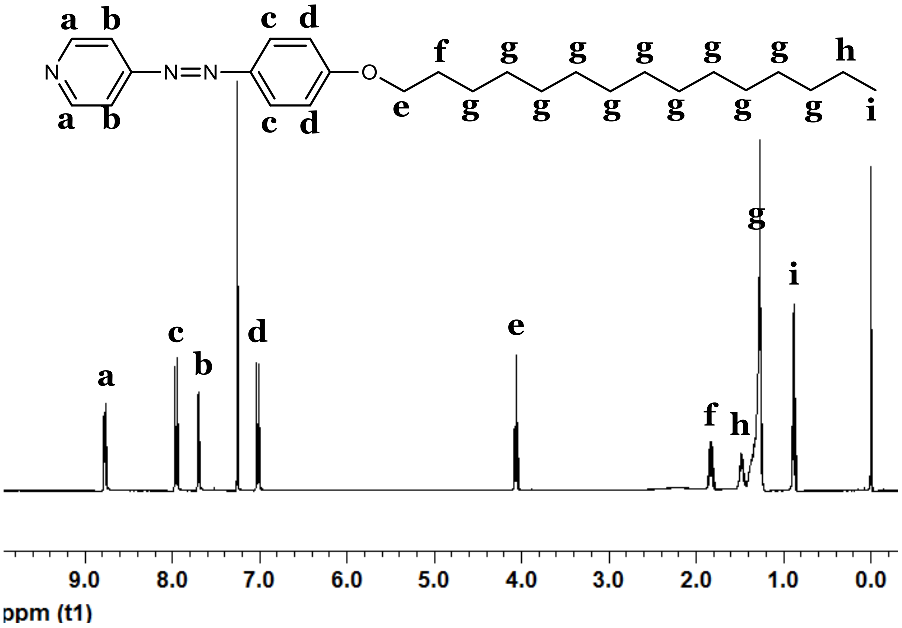

Figure S1 ^{1}H NMR spectrum of the A15AzPy (compound a2).

1.3 Synthesis of halogen–bonded complexes (compound a3)

Bromine (0.0799 g, 0.5 mmol) or iodine (0.127 g, 0.5 mmol) dissolved in hexane (25 mL), respectively, then directly added to a solution of azopyridine compounds (0.5 mmol) in chloroform (5 mL). The contents were stirred for 20 h at room temperature. The jacinth precipitate was filtered and dried.

1.4 Synthesis of 15X@QDs (compound a4)

Oleic acid-modified core-shell ZnCdSe/ZnS QDs (solvent: *n*–hexane) were purchased from Wuhan Jiayuan Quantum Dot Co., Ltd. The maximum emission wavelength is 625 nm ± 5 nm, and the size is 5–10 nm. The 15X@QDs was prepared by dissolving halogen-bonded 15X (X: Br or I) complexes (0.4

wt%) in THF, then added QDs into 15X solution and stirred 5 min evenly at room temperature. The fibrous materials were spontaneously formed upon evaporation of one drop of the resultant solution on the surface of glass substrates at room light, and the fibrous morphologies can be stably stored for at least half a year.

2. The Optical images of bromine bonded fibres

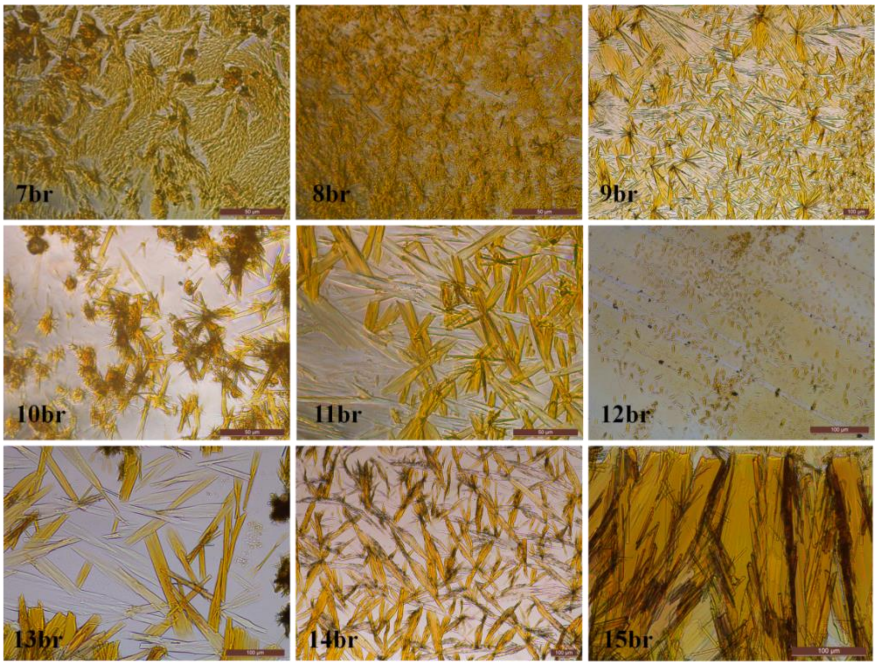

Figure S2 Optical images of bromine bonded fibres with different alkyl chains formed in THF.

3. The images of 15Br fibres with different mass concentration formed in THF

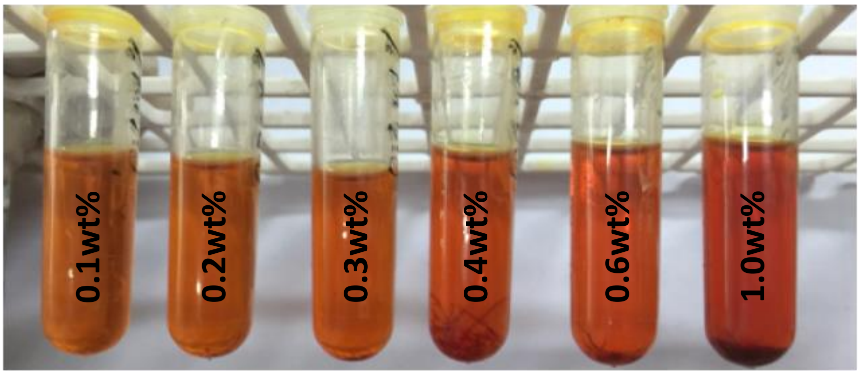

Figure S3 The picture of different mass concentration of 15Br from 0.1 to 1.0 wt% in THF.

4. The self–assembled morphologies of 15Br with 0.4 wt% mass

concentration obtained in different solvents at room temperature

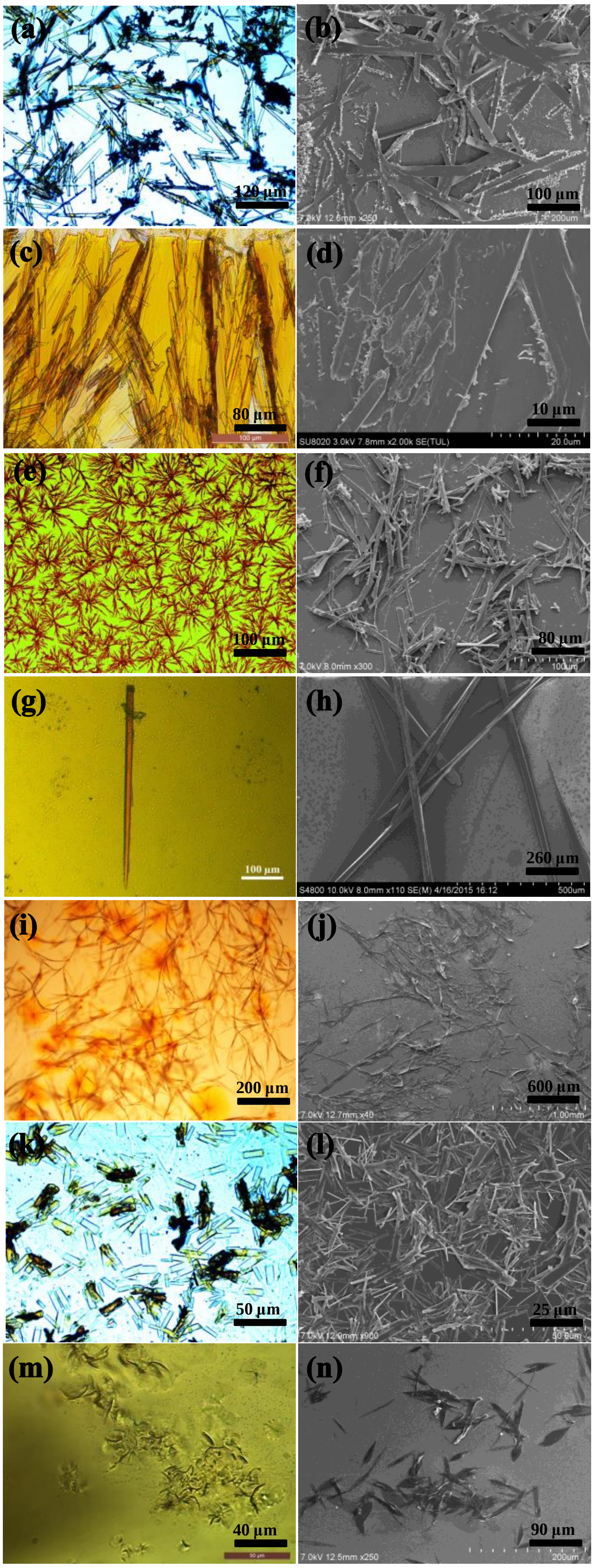

Figure S4 Optical and SEM images of the self-assembled fibres formed from 15Br (0.4 wt %) in different organic solvents at room temperature. (a) dichloromethane (DCM), (c) tetrahydrofuran (THF), (e) ethanol, (g) acetone, (i) N,N−dimethylformamide (DMF), (k)

methanol and (m) dimethylsulfoxide (DMSO) are optical images, (b, d, f, h, j, l, n) are SEM images.

In these solvents, the halogen bonded fibres appeared with the diameters of micron range in DCM (P=3.1), THF (P=4.2), ethanol (P=4.3), acetone (P=5.4), DMF (P=6.4), methanol (P=6.6) and DMSO (P=7.2), as shown in Fig. S4. Different nature of the solvents gives rise to different morphologies of the self-assembled fibres. The morphologies consisted of bundles of halogen bonded fibres with a small length-diameter ratio from DCM, ethanol and methanol resemble branches (Fig. S4(a), (e), (k)). By contrast, a big length-diameter ratio of 15Br fibres was observed in THF, acetone and DMF, respectively (Figs. S4(c), (g), (i)). The bamboo leaves-like geometries were obtained in DMSO (Fig. S4(m)) with a large polarity. These results suggest that the nature of organic solvents has an important influence on the fabricated morphologies of halogen bonded fibres. However, the pristine molecule A15AzPy formed lamellar crystal instead of self-assembled fibres in those solvents as shown in Fig. S5.

5. **The self–assembled morphologies of A15AzPy with 0.4 wt% mass concentration obtained in different solvents at room temperature**

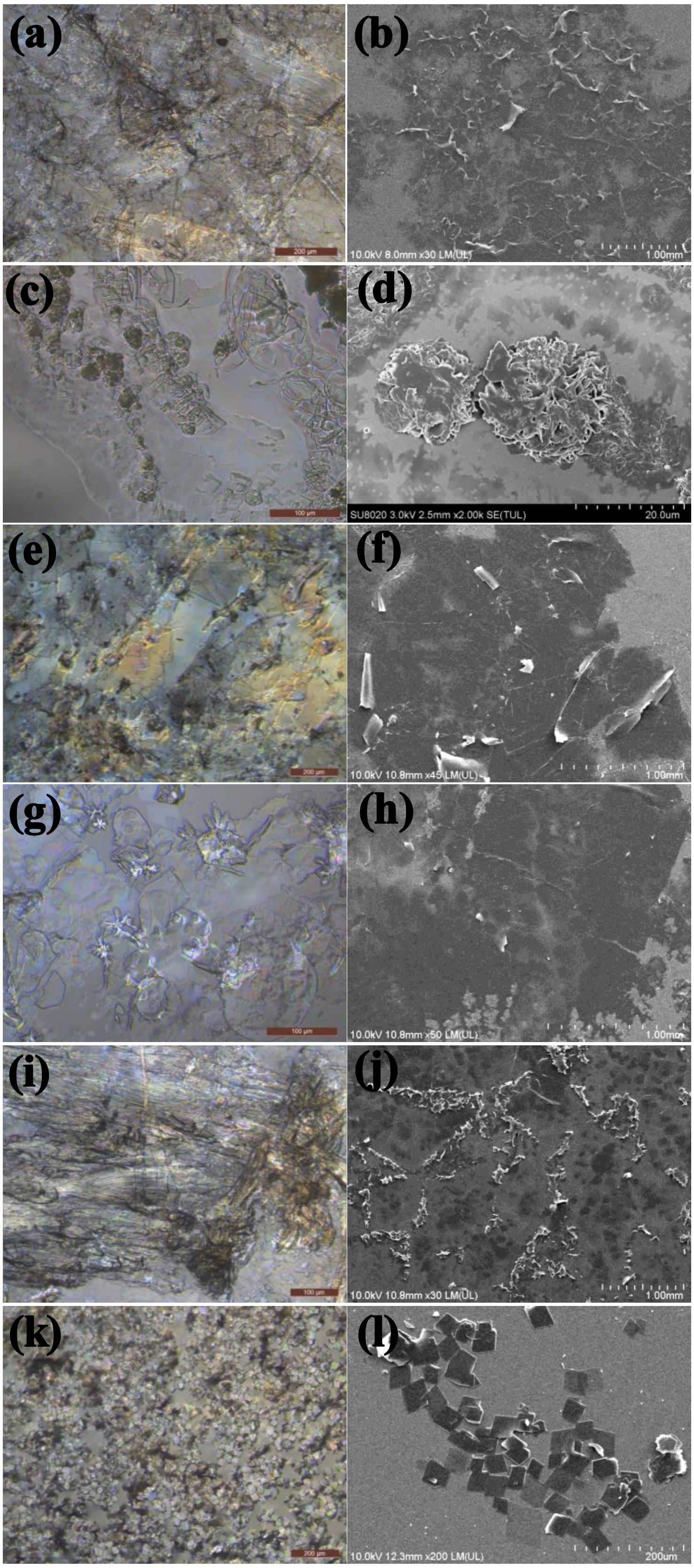

Figure S5 Optical and SEM images of A15AzPy with 0.4 wt% mass concentration obtained in different solvents at room temperature. (a) dichloromethane (DCM), (c) tetrahydrofuran (THF), (e) ethanol, (g) N,N-dimethylformamide (DMF), (i) methanol and (k) dimethylsulfoxide (DMSO) are optical images, (b, d, f, h, j, l) are SEM images.

As shown in Fig. S5, The A15AzPy could not form self–assembled fibres in these six different organic solvents.

6. The DSC and XRD of 15Br and 15Br fibres

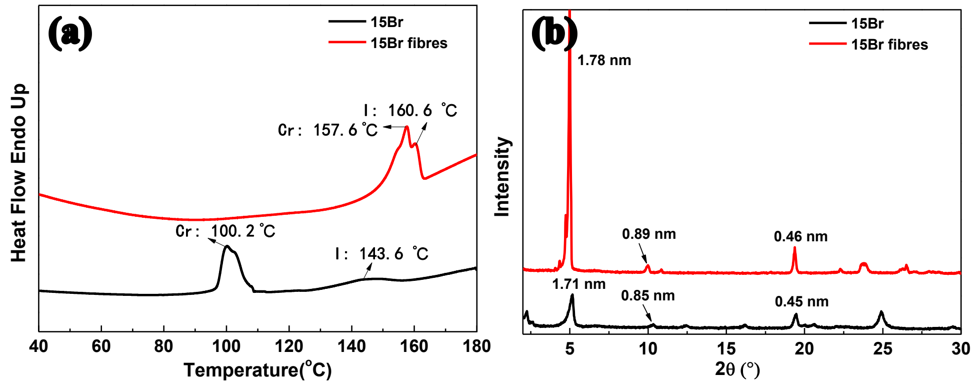

Figure S6 DSC measurements (a), XRD patterns (b) of 15Br and 15Br fibres at room temperature.

7. Laser scanning confocal microscopy images of supramolecular fibres

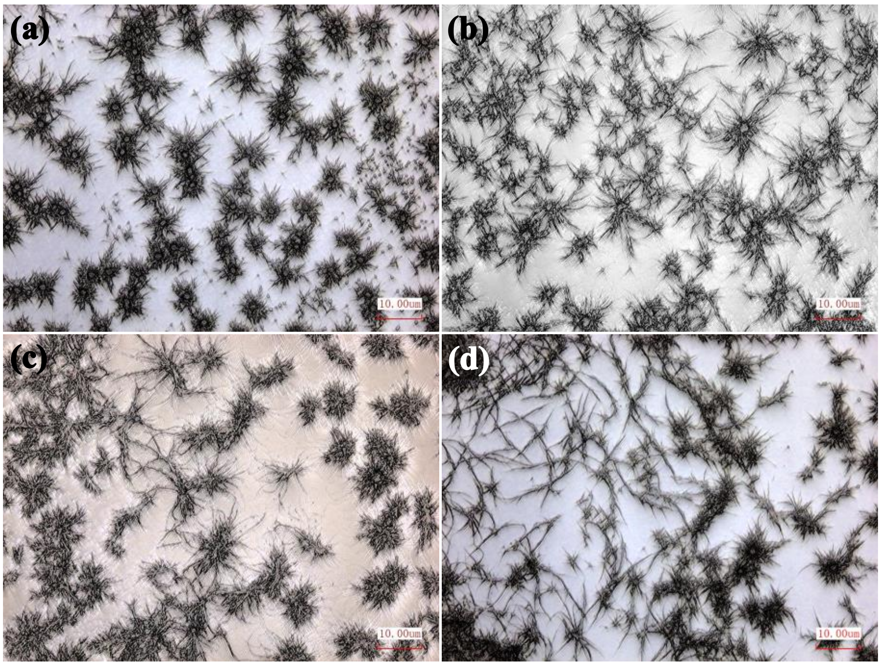

Figure S7 Laser scanning confocal microscopy images of supramolecular fibres from the mixtures of 15Br (0.4 wt%) in THF and ZnCdSe/ZnS QDs in *n*-hexane. The volume ratio of 15Br and QDs are (a) 3:1, (b) 2:1, (c) 1.5:1, (d) 1:1.

8. SEM and EDS images

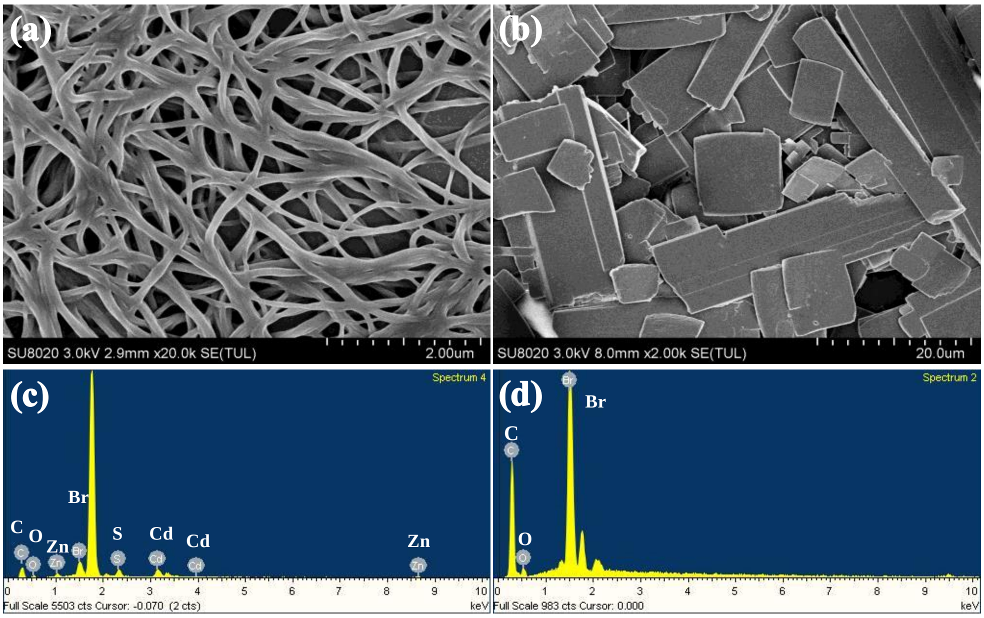

Figure S8 SEM images of 15Br (0.4 wt%) in THF (a) adding QDs, (b) adding *n*–hexane mixed solvent, (c) and (d) are their EDS images.

9. Optical microscopy of A15AzP@QDs

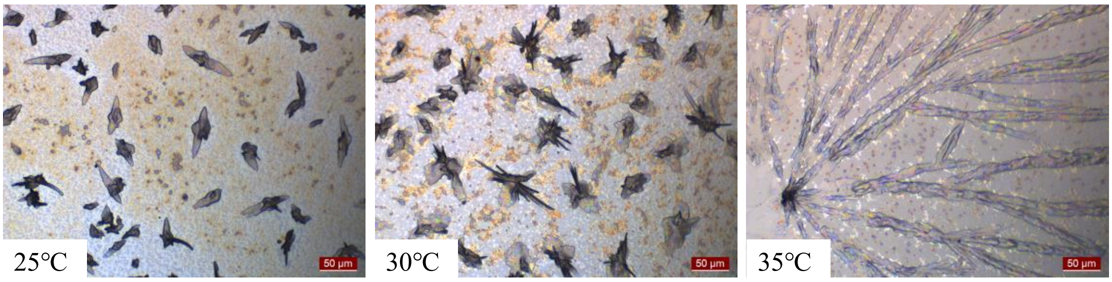

Figure S9 Optical images of the self-assembled fibres formed from A15AzPy@QDs (volume-to-volume ratio = 3:1) in THF at different temperature.

Although the A15AzPy could not form self-assembled fibres, but A15AzPy@QDs could form self-assembled fibres due to the van der Waals interactions between alkyl chains. As the temperature increases, we observed the process of self-assembled fibre growth from small crystal to fibres.

10. Polarizing optical microscopy of 15Br and 15Br@QDs

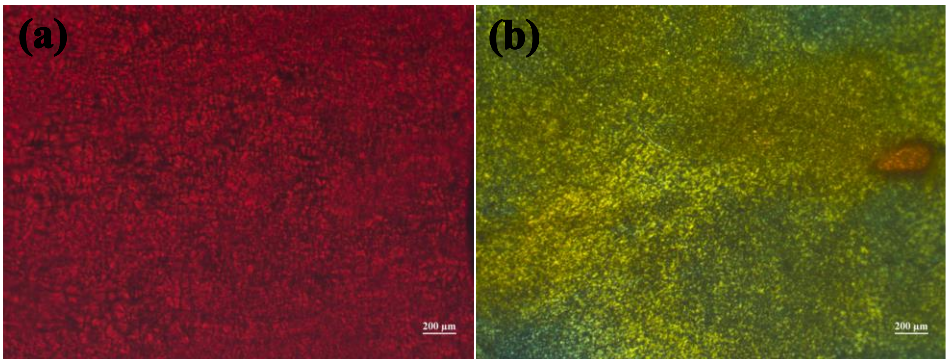

Figure S10 POM pictures of 15Br at 125 °C (a) and 15Br@QDs at 152 °C (b) on cooling from the isotropic phase.

11. Polarized UV-vis spectra

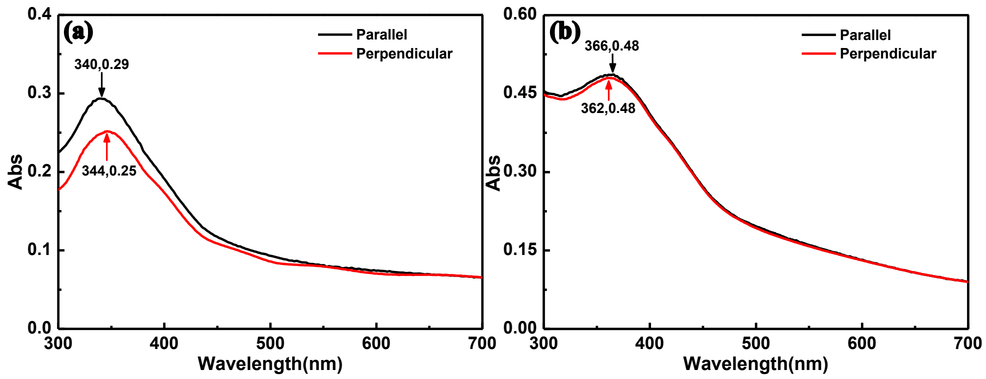

Figure S11 Polarized UV–vis spectra of 15Br@QD (a) and 15Br (b) films. The black and red curves are the absorption parallel and perpendicular to the orientation direction, respectively.

Moreover, the aligned mesogens 15Br@QD show strong anisotropy in their polarized UV–vis absorption spectra and the orientation factor is 0.052 compared favorably to the pristine 15Br (0.004).

12. Standard deviation of the aspect ratio

After mixing with QDs, the aspect ratio of the fibres is different. To describe the scattering degree of measured values, standard deviations could be calculated.

The equation is shown as follow:

$$S=\{〔n\sum x^2-(\sum x)^2〕/〔n(n-1)〕\}^{1/2}$$

For the 15Br and 15Br@QDs, the standard deviations are 20.88 and 8.44, respectively. In addition, the standard deviation of the 15I@QDs is 20.80. The results suggest that the QDs can enhance the dispersion uniformity of the 15Br fibres. The 15I@QDs fiber distribution is more randomness in comparison with the 15Br@QDs.

Notes and references

1. Chen, Y.; Yu, H.; Zhang, L.; Yang, H.; Lu, Y. Photoresponsive liquid crystals based on halogen bonding of azopyridines. *Chem. Commun.* **2014**, *50*, 9647–9649.
